# Cell-Based Computational Models of Organoids: A Systematic Review

**DOI:** 10.3390/cells15020177

**Published:** 2026-01-19

**Authors:** Monica Neagu, Andreea Robu, Stelian Arjoca, Adrian Neagu

**Affiliations:** 1 Department of Functional Sciences, “Victor Babeș” University of Medicine and Pharmacy of Timisoara, E. Murgu Sq., No. 2, 300041 Timisoara, Romania; neagu.monica@umft.ro (M.N.); arjoca.stelian@umft.ro (S.A.); 2 Center for Modeling Biological Systems and Data Analysis, “Victor Babeș” University of Medicine and Pharmacy of Timisoara, E. Murgu Sq., No. 2, 300041 Timisoara, Romania; 3 Department of Automation and Applied Informatics, Politehnica University of Timisoara, 300006 Timisoara, Romania; andreea.robu@aut.upt.ro; 4 Department of Physics and Astronomy, University of Missouri, Columbia, MO 65211, USA

**Keywords:** organoid growth, agent-based models, in silico models

## Abstract

Organoids are self-organizing multicellular structures generated in vitro that recapitulate the micro-architecture and function of an organ. They are commonly derived from stem cells but can also emerge from pieces of proliferative tissues. Organoid technology has opened novel ways to model development and disease, but it is not without challenges. Computational models of organoids have been established to elucidate organoid growth and facilitate the optimization of organoid cultures. This article is a systematic review of in silico organoid models constructed at single-cell or subcellular resolution. PubMed, Scopus, and Web of Science were searched for original papers published in peer-reviewed journals before 26 September 2025, yielding 439 records after deduplication. Two independent reviewers screened their titles and abstracts, retrieved 84 papers for full-text scrutiny, and identified 32 papers that met the inclusion criteria. They were grouped by organoid type: 12 intestinal, 1 airway, 2 pancreas, 3 neural, 1 kidney, 1 inner cell mass, 9 tumor, and 3 generic. The analysis of these works revealed that computer simulations guided experimental work. Parsimonious computational models provided insights into diverse organoid behaviors, such as the rotation of airway organoids, size oscillations of pancreatic organoids, epithelial patterning of neural tube organoids, or nephron segment formation in kidney organoids. Generally, a deep understanding was achieved through combined in silico and in vitro investigations (e.g., optic cup morphogenesis). Recent research trends suggest that next-generation computational models of organoids may emerge from a more detailed understanding of the complex regulatory circuits that govern stem cell fate, and machine-learning-based, high-throughput imaging of organoids.

## 1. Introduction

Organoids are multicellular structures that closely resemble the composition and function of in vivo organs. They are derived from stem cells or tissue-resident progenitor cells cultured in a three-dimensional (3D) environment, in media supplemented with a cocktail of growth factors [1,2,3]. Organoid types established to date include cerebral, retinal, inner ear, intestinal, pancreatic, gastric, liver, kidney, airway, lung, heart, mammary gland, prostate, and skin [4]. Their shape is organ-specific, but different from the shape of the respective organ, because the cells self-organize in the absence of their native environment [1]. 

The term “organoid” was coined by Smith and Cochrane in their case study of a cystic teratoma [5], but the roots of the field are older. Ross G. Harrison invented the hanging drop method for culturing 3D embryonic tissue fragments in 1906 [6]. A year later, Henry Van Peters Wilson discovered that dissociated cells from silicious sponges self-organize into clusters of organotypic arrangement [7]. Other investigators observed similar phenomena in different species [8]. In the early 1960s, Steinberg proposed the differential adhesion hypothesis to explain the reaggregation of dissociated tissues and subsequent cell sorting [9,10,11]. His theory was incorporated into computational models that recapitulated certain morphogenetic processes observed in vivo and in vitro [12,13,14,15,16]. Decades of research on 3D tissue culture clarified the impact of the extracellular matrix (ECM) on multicellular self-organization [17,18]. Nevertheless, organoid research has gathered momentum over the past 15 years due to four major advancements in cell biology: (i) the emergence of reliable methods of embryonic stem cells culture, (ii) the ability to reprogram somatic cells to create induced pluripotent stem cells, (iii) the development of high-throughput techniques to characterize the heterogeneity of cell populations via massively parallel single-cell RNA sequencing, and (iv) progress in genome engineering by using CRISPR-Cas9 [19]. 

Besides providing insights into physiological mechanisms, organoids were successful in disease modeling [1], including cancer [20]. It is well established that in vitro models provide valuable insights into cancer biology and drug response. Their accuracy, however, varies depending on their ability to recapitulate the composition and architecture of the in vivo tumor and its microenvironment. Indeed, successful drug tests conducted on 2D cultures often failed in clinical trials, motivating the emergence of 3D cancer models [20]. Some of them were obtained by seeding cells on porous scaffolds, whereas others were scaffold-free, such as spheroids and organoids. Spheroids are obtained from the aggregation of single-cell suspensions and subsequent self-organization. They can be built from established cancer cell lines as well as from dissociated tumor tissue and can also include non-malignant cell types commonly found in the tumor microenvironment (TME). In contrast, tumor organoids are obtained by growing mechanically and enzymatically dissociated tumor tissue in hydrogels that contain basement-membrane matrix components. They emerge by the proliferation and self-organization of cancer cells, adult stem cells, cancer stem cells, and other cell types present in the cancer biopsy samples [21]. Surprisingly, tumor organoids may be overgrown by healthy organoids if the initial sample includes healthy tissue. Therefore, selective culture conditions are needed to establish pure tumor organoid cultures [20]. So far, several types of cancer organoids have been derived from tumor biopsies, and they have been found to recapitulate the patient’s response to anticancer drugs [22].

Despite remarkable results in disease modeling, drug screening, and regenerative medicine [23], organoid research faces considerable challenges [24]. Typical organoids range from tens of micrometers to several millimeters in size. As they grow, the efficiency of nutrient supply and waste removal decreases, ultimately affecting their lifespan. Also, organoid populations are often heterogeneous in terms of shape and function because the initial conditions (substrate, starting cell population) can vary, and organoid development also involves stochastic processes. Therefore, organoid research is believed to benefit from engineering approaches to organ-on-a-chip systems [24] and theoretical modeling [25,26,27,28]. 

Mathematical and computational models of multicellular systems have been used for decades to elucidate the molecular and cellular mechanisms underlying in vivo morphogenesis and disease progression [13], and to test working hypotheses in tissue engineering [16]. The growing primary literature stimulated the publication of several review articles [25,26,27,29]. They provided new insights and built connections between models, which were highly needed, given the methodological differences between the original studies. 

To our knowledge, the literature lacks systematic reviews of computational models of organoids; this is the gap the present paper aims to fill. The main questions addressed by this review are whether computational models of organoids can sort out morphogenetic mechanisms involved in organoid growth and whether they can generate knowledge beyond that established by experiments. 

## 2. Methods

This review was conducted according to the reporting guidance provided by the Preferred Reporting Items for Systematic Reviews and Meta-Analyses (PRISMA) 2020 statement [30]. 

### 2.1. Eligibility Criteria

Studies included in this review met all the following criteria: original articles published in peer-reviewed journals, written in English, and dealing with computational models of organoids that describe state changes and rearrangements of the constituent cells. In the literature, such models are referred to as cell-based, particle-based, or agent-based models. Non-English articles and grey literature (e.g., preprints, conference proceedings, and theses) were excluded. 

### 2.2. Information Sources and Search Strategy

We conducted a systematic search on PubMed, Scopus, and Web of Science to identify relevant papers as of 26 September 2025. Titles and abstracts were searched in each database, whereas for Scopus, keywords were also included. The Boolean expressions of search terms are listed in the Supplementary Material, Table S1. 

### 2.3. Document Selection

Search results were exported in comma-separated values (CSV) format and analyzed in Excel (Microsoft Corporation, Redmond, WA, USA). Duplicates of documents with a digital object identifier (DOI) were identified and removed using Excel. In the absence of a DOI, duplicates were manually spotted and eliminated. 

Two independent reviewers screened each record based on their title and abstract. If both reviewers deemed a record potentially eligible, the full text was retrieved and assessed for eligibility by each reviewer. In case of a disagreement during abstract screening, the full text was retrieved, and a third reviewer was also asked to evaluate the paper. The final decision was taken in meetings attended by all three reviewers. 

### 2.4. Data Collection

Eligible papers were studied independently by two reviewers to extract the following data items: organoid type, in silico model class, resolution, ECM representation, description of cell differentiation and proliferation, simulation of multicellular system dynamics, and model validation. 

## 3. Results

**Figure 1:**
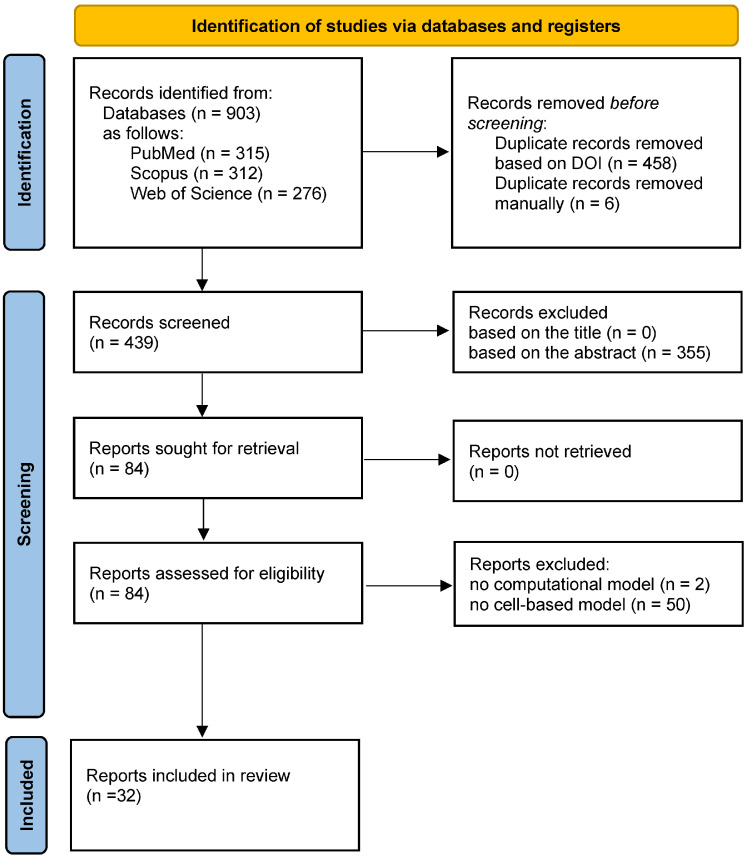
PRISMA 2020 flow diagram showing the stages of literature handling.

Figure 1 shows the PRISMA 2020 flow diagram of document identification and screening [30]. 

This section provides an overview of the original articles that satisfy the inclusion criteria. To highlight common features in different models, the information is organized into subsections by organoid type. 

### 3.1. Models of Intestinal Organoids

The small intestine protects us from the harsh chemicals and bacteria present in its lumen while enabling the uptake of nutrients and water. To ensure a large area for absorption, the small-intestinal epithelium features villi, finger-like projections into the lumen, mainly covered by enterocytes—non-proliferating cells specialized in absorbing intestinal content [31]. Between villi, there are invaginations, known as crypts, whose bottoms host intestinal stem cells (ISCs) intercalated between Paneth cells (PCs); the latter secrete Wnt and Notch ligands (to preserve the stemness of adjacent ISCs), epidermal growth factor (EGF, to support stem cell proliferation), and antimicrobial agents. The lateral walls of the crypts are lined by transit-amplifying (TA) cells (which divide 2–5 times before maturation), and differentiate into enterocytes (ECs), goblet cells (GCs—produce mucus), enteroendocrine cells (EECs—secrete hormones), and tuft cells (involved in immune response). As ISCs and progenitor cells divide within the crypt, differentiated cells are pushed upward along the adjacent villus; within a few days, as they reach the tip, they undergo anoikis (programmed cell death caused by loss of anchorage) and end up in the lumen. Thus, the stem cell niche residing in crypts ensures the rapid turnover of the intestinal epithelium [31]. 

The cell-based computational model of Buske et al. described the self-organization of small intestinal crypts [32]. In their model, cells were represented as sticky elastic spheres, thereby considering forces generated by cell adhesion and deformation. Their model describes lineage specification of undifferentiated cells into PCs, ECs, and GCs. Cells with high Wnt and Notch signaling are considered to retain their stemness. Undifferentiated cells with high Wnt but low Notch activity become primed for the PC lineage, whereas at low Wnt but high Notch signaling ISCs are assumed to be specified into the EC lineage. EC progenitors become primed for adopting GC characteristics if Notch activity also falls below a threshold. It is further assumed that PC progenitors actively migrate down the crypt, whereas other primed cells move in the opposite direction. In the model, active migration is driven by an externally applied uniform force field. A cell moves under the action of the vector sum of these cell-type-dependent migration forces and all the forces associated with its interaction with other cells and the basal membrane [32]. 

Priming is considered reversible: transitions are allowed between different progenitor cells as well as from progenitor cells to ISCs. A primed cell can also attain a terminal differentiation state. For example, EC and GC progenitors become irreversibly differentiated and stop dividing if Wnt signaling drops below a certain threshold, which happens as they migrate beyond the crypt-villus junction. PC progenitors are assumed to reach terminal differentiation at high Wnt signaling as soon as they finish their current cell cycle. 

Buske et al. assumed that Wnt activity is favored by a high curvature of the monolayer, which might be explained at the molecular level by integrin signaling [32]. Indeed, in a highly curved epithelium, a large proportion of the cell surface is connected through integrins to the basal membrane. 

Thalheim et al. simulated the long-term dynamics of the cell population of intestinal crypts using the model elaborated by Buske et al. [33]. Their results provided insights into the clonal competition underlying normal tissue homeostasis, as well as the impact of perturbations of Wnt and Notch signaling observed in colorectal cancer. 

Single ISCs isolated from crypts could be expanded in Matrigel [34], and gave rise to intestinal organoids comprising all the cell types encountered in the small-intestinal epithelium [35]. Building on their intestinal crypt model, Buske et al. went on to develop the first computational model of intestinal organoids [36]. Therein, an essential novelty was the in silico representation of the basal membrane as a triangulated, flexible polymer network. In its minimum energy state, this network has a preferential angle between adjacent triangle normals. Therefore, by default, the polymer matrix adopts a spherical shape, but interactions with the cell monolayer can distort it. The mesh size is controlled to accommodate a growing cell population, and new triangles are inserted whenever the area of a triangle exceeds a threshold. 

As the attached cells grow, proliferate, and migrate, they remodel the polymer network, inducing shape changes observed in experiments, such as the budding that initiates the formation of crypt-like domains. It is assumed that PCs exhibit stronger adhesion to the polymer network than ISCs, resulting in increased curvature of the epithelial monolayer in regions where both cell types are present. Mature cells are subsequently displaced from the crypt-like structures and eventually detach from the polymer matrix, suffering anoikis. Their remnants are modelled as inert, deformable particles that cannot grow or adhere to other components. Nevertheless, as they accumulate, they generate internal pressure (referred to as cyst pressure), which can ultimately lead to organoid rupture [36].

The growth patterns of intestinal organoids were further explored by Thalheim et al. [37]. To facilitate bud formation, they extended the computational model of Buske et al. [36] with a polymer network attached to the apical side of the cell monolayer. This apical net mediates cell–cell interactions beyond first-order neighbors. The simulations illustrated the influence of Wnt and Notch signaling on the organoid’s cellular composition and reproduced the experimentally observed branched and cyst-like phenotypes. Although the model was unable to account for the shape changes of cells associated with cyst-like growth, a geometric transformation of the model system provided excellent agreement with in vitro results [37]. 

To simulate intestinal crypt dynamics, Pin et al. built a model of granular material evolving on a 1D helical path that runs along the 3D wall of the crypt, akin to a pearl necklace tightly wound around a test tube [38]. In their work, the crypt’s base is represented by a spiral that covers a hemisphere, whereas its body is modeled as a helix. Cells are viewed as grains whose state changes are described by the transcriptional pathways that control ISC maintenance and differentiation. The 1D chain, however, is not considered rigid: at each time step of the Monte Carlo simulation, the spiral and helix are reshaped according to cell growth, proliferation, or death. Cell growth is considered isotropic. Along the crypt axis, it determines an upward migration, whereas in the transversal plane, it causes an increase in the helix diameter. Remarkably, the model predicts a realistic distribution of all the cell types hosted by intestinal crypts without assuming active migration forces. The localization of PCs at the crypt base results from their inability to divide and the structural relaxation of the heterotypic granular system. Wnt signaling in a given position is assumed to only depend on the local distribution of PCs, goblet-Paneth progenitors, ISCs, and transit-amplifying cells [38]. 

An extension of the granular material model [39] provided interesting insights into the mechanism of intestinal crypt fission observed both in vivo and in intestinal organoids [35]. Crypt fission, whereby a single crypt divides into two daughter crypts, is a physiological phenomenon involved in intestinal growth and maintenance. The extended model treats mature cells (e.g., PCs) as viscoelastic bodies (Bingham plastics) and proliferative cells (e.g., ISCs) as Newtonian fluids. The hypothesis that PCs are stiffer than ISCs was confirmed by atomic force microscopy, and simulations performed under this hypothesis demonstrated budding in domains where ISCs were surrounded by PC-rich patches [39], as observed in intestinal organoids as well as in the mouse small intestine [40]. 

A similar mechanism was found at play in a 2D simulation of intestinal organoids using an off-lattice cell-center model from the Chaste software [41,42,43] (https://chaste.github.io/, accessed 16 July 2025). Interactions are described by elastic forces acting between adjacent cell centers, and cell shapes result from Voronoi tessellation. In the model of Langlands et al. [40], the organoid’s cross-section is represented by a chain of cells lining a lumen that is embedded in a mass of non-epithelial cells, which represent the Matrigel. The simulations demonstrated that in regions with a relatively high proportion of PCs, the softer ISCs are pushed out of the monolayer and into the non-epithelial cell mass, thereby initiating the formation of new crypt-like domains. Experiments revealed that differences between PCs and ISCs in terms of cell–basement membrane adhesion are also important for budding. Indeed, organoids grown in the presence of a β4 integrin blocking antibody exhibited fewer fission events [40]. 

Almet et al. conducted extensive simulations using the cell-center model due to Langlands et al., and found that budding is favored by PCs having both higher stiffness and stronger cell–basal membrane adhesion than ISCs [44]. The model was further extended by Montes-Olivas et al., who also considered TA cells to account for the presence of proliferative stiff cells in intestinal organoids, and ECs to represent differentiated cells produced by TAs [45]. They modified the cell cycle diagrams accordingly and inferred model parameters from single-cell RNA sequencing data of the murine small intestine [46]. Although their model did not incorporate the signaling pathways that regulate cell differentiation, it reproduced the mean number of crypts observed in experiments. Notably, the authors devised a crypt counting algorithm applicable to both brightfield microscopy snapshots of organoids and configurations obtained from computer simulations [45]. 

Yang et al. scrutinized potential factors that bring about shape changes in intestinal organoids [47]. Inspired by computational vertex models, they built an analytical theory to describe the stationary state of an organoid composed of a crypt-like and a villus-like region. For simplicity, the overall shape of the organoid was approximated by two spherical caps connected by a thin neck, and the free energy of the entire system was expressed in terms of cap radii, cell volume, epithelial sheet thickness, and surface tensions associated with the apical, lateral, and basal portions of the cell membrane. The theoretical model predicted that different budding mechanisms would lead to distinct organoid morphologies—e.g., ratios of epithelial thickness or curvature in crypts versus villi. Experiments consistently showed that the epithelial layer is more curved and thicker in the crypts than in villi, which agreed with a single theoretical scenario, that bulging and subsequent budding occur through differential spontaneous curvature (i.e., spontaneous tissue bending). Furthermore, the model predicted that budded crypts preserve their shape despite lumen inflation, whereas bulged crypts open up, as observed in vitro [47]. 

The morphology and biomechanics of human colon organoids were investigated by Laussu et al. in the framework of an active vertex model combined with finite element modeling [48]. The in silico organoid’s equilibrium shape was obtained via energy minimization, whereas cell-level strains elicited by certain types of imposed stress (external load, active contraction, and osmotic pressure) were computed using the finite element model. 

Besides gene expression, chemical cues, and cell biomechanics, tissue self-organization is also influenced by the mechanical properties of the ECM. Elosegui-Artola et al. explained the symmetry breaking of multicellular spheroids and morphological evolution of intestinal organoids based on cell proliferation, cell motility, tissue viscosity, and ECM viscoelasticity [49]. They cultured organoids in alginate-Matrigel matrices that differed in stiffness and viscoelasticity; the latter was controlled by the molecular weight of the alginate and crosslinker concentration, while the Matrigel concentration was kept constant. In elastic matrices, organoids grew slowly and remained spherical. By contrast, in viscoelastic matrices, they grew rapidly and exhibited morphological instability (finger formation) and in vivo-like cell differentiation and patterning. 

Experimental findings were replicated by a minimal computational model of the overdamped dynamics of cells represented by soft elastic spheres subject to intercellular forces, cell–ECM repulsion, and random motility [49]. The ECM is represented by a set of cell-sized beads moving passively in a viscous liquid under the action of three types of forces that account for matrix elasticity and remodeling. First, each bead is tied to its initial position by a harmonic spring that pulls it back, provided that the displacement is less than a bead radius; otherwise, the bead is assumed to break away from its initial position and establishes a new equilibrium position (to which it is tied again by an elastic spring). Second, beads interact by short-range repulsion and mid-range attraction (acting along two bead diameters). Third, beads are repelled by adjacent cells. In the model, the organoid’s cross-section is described as a chain of cells interacting via a harmonic spring potential and a bending potential that tends to minimize the chain’s curvature. Cell division is attempted at a fixed rate by placing a daughter cell next to the proliferating cell, and a Monte Carlo algorithm decides whether an energetically unfavorable cell division attempt is accepted or not. Thus, cell proliferation depends on the local stress. Just as in experiments conducted in the presence of a focal adhesion kinase inhibitor, budding did not occur in simulations performed in the absence of cell motility. The same outcome was observed in the absence of cell proliferation. In silico, symmetry breaking mainly occurred in regions with elevated cell motility and/or proliferation rate [49].

To assess the impact of signaling pathways on the compartmentalization of the intestinal epithelium, Larrañaga et al. seeded organoid-derived single cells onto 2D substrates coated with freeze-dried reconstituted basement membranes and observed that the cells grew into a self-organized monolayer featuring crypt- and villus-like domains [50]. Then, they loaded the basement membrane with exogenous Wnt3a in dots approximately 0.1 mm in diameter, arranged in a square lattice. Remarkably, the micropatterns dictated the size and shape of the crypt-like regions. To decipher the interplay between endogenous and exogenous Wnt signaling and the cellular arrangement, they developed a cell-based computational model using the ya||a platform [51] (https://github.com/germannp/yalla, accessed 7 October 2025). Previous investigations of intestinal organoid monolayers grown on soft hydrogels revealed that the size of the crypt-like domain depends on the substrate stiffness and cell traction forces [52]. Such planar cultures of intestinal organoids are appealing since they allow for time-lapse force mapping at subcellular resolution and enable the visualization of collective cell migration. Pérez-González et al. constructed a 3D vertex model, which was found capable of replicating the experimentally observed monolayer morphologies on both hard and soft substrates, although it neglected cell rearrangements. Experiments and simulations indicated that crypt-like compartments bend due to apical constriction, pushing the underlying matrix [52]. 

Taken together, the computational studies of intestinal organoids elucidated their growth patterns and cellular composition, albeit with different assumptions. For example, 3D models suggest that the confinement of PCs to the bottom of crypt-like domains requires active cell migration, whereas low-dimensional models that represent the epithelium as a chain of cells can explain PC distribution without such an assumption. Also, in silico models clarified the role of cell–ECM interaction in organoid development. 

### 3.2. Model of Airway Organoids

Seeking to model several pulmonary conditions, Sachs et al. established airway organoids (AOs) [53]. They obtained epithelial cells from lung tissue samples of non-small-cell lung cancer patients, embedded them in basement membrane extract (BME), and manipulated signaling pathways relevant to airway epithelium. Within a few days, AOs emerged in the form of hollow spheroids whose cellular composition and stratification recapitulated that of human airways. Basal cells lined their surface, in contact with the BME, whereas club cells, secretory cells, and multi-ciliated cells formed a mixed layer next to the fluid-filled lumen. AOs were functional (beating cilia and mucus production could be visualized by time-lapse microscopy) and expressed airway cell type-specific genes. They could be passaged for more than a year without a decline in proliferation rate. AOs were used for disease modeling, including cancer, cystic fibrosis, and respiratory syncytial virus (RSV) infection. 

RSV-infected organoids displayed an intriguing motility: they rotated and migrated within the BME and coalesced when they encountered one another. To clarify the underlying mechanisms at the cellular level, Sachs et al. created a mathematical model comprising point particles (which represent basal cell centers) constrained to move on the surface of a sphere while being connected by springs [53]. Each cell is endowed with a polarity vector that points along the traction force generated by actomyosin contractility [54]. 

Typically, the polarity vector changes direction stochastically over time. In an isotropic environment, cells display a persistent random walk, wherein they retain their direction of polarization for a while and then reorient themselves. The average time over which the intrinsic polarity is maintained is known as the persistence time. 

In a tissue, cell movement is constrained by interactions. The total force acting on a cell is the resultant of the traction force and the forces of adhesion exerted by its neighbors, modeled by spring forces. 

The model reproduced the observed coordinated cell movements only if the stochastic dynamics of the polarity vectors were modulated deterministically by cell–cell communication such that each cell tended to change its intrinsic polarity to align with the direction of the total force acting on it. The emergence of organoid rotation was found to be favored by increased persistence time [53]. 

### 3.3. Models of Glandular Organoids

Hof et al. cultured mouse pancreas-derived organoids and visualized their dynamics using bright-field microscopy [55]. The organoids were monocystic, roughly spherical, and exhibited growth heterogeneity and size oscillations. They hypothesized that the organoid’s volume increases due to (i) a growing internal pressure because the cells release osmotically active substances into the lumen, and (ii) cell proliferation in the organoid shell. When cell division cannot keep up with the organoid’s osmotic swelling, certain cell–cell bonds break, releasing part of the luminal content; the pressure drops, and the cells reconnect, sealing the leak. This scenario was tested using an off-lattice, center-based mathematical model. 

Just as in [53], the organoid shell is modeled as a triangulated network of point particles interacting via harmonic springs while also generating stochastic traction forces. When a cell divides, a new point is inserted into the center of an adjacent triangle. To maintain the spherical shape of the growing shell, a bending energy is considered between neighboring triangles, which is minimal when the triangles are aligned. The organoid’s dynamics are simulated by solving Newton’s equation of motion in the overdamped limit (i.e., when the cell velocity is proportional to the resultant force). Leakage occurs whenever the average distance between adjacent cells exceeds a threshold. 

The model predicts that the frequency of size oscillations depends both on organoid size and cell division dynamics. Simulations indicate, in agreement with experiments, that leakage is more frequent in small organoids. Also, the periods of size oscillations correlate with proliferation patterns: no ruptures occur while the number of cells grows exponentially, but they happen periodically during the linear growth of the cell population [55]. 

Mouse pancreatic organoids were modeled beyond the spherical growth phase by Dahl-Jensen et al. [56]. They took cells from pancreatic buds at embryonic day 10.5 (E10.5) and cultured them in Matrigel. Initially, the seeded cells expanded in a spherical arrangement and then turned into a branched structure resembling an embryonic pancreas. The authors built a cellular automaton model on a 3D cubic lattice. In the model, a grid point is occupied by organoid biomass or medium. The biomass attempts to expand into adjacent medium-filled grid points (6 nearest neighbors and 12 next-nearest neighbors). Site occupancy is derived from a continuous function whose evolution is governed by the concentration of a signaling molecule that can either promote or inhibit growth. The latter is produced by cells, diffuses freely, and degrades over time. The simulated organoid architectures replicated their in vitro counterparts if the signaling molecule was an inhibitor capable of forming dimers (Hill coefficient, *H* = 2) or trimers (*H* = 3)—potential candidates include Notch inhibitors. The model suggests that a strong and local inhibitor field drives the observed stochastic branching [56]. 

In conclusion, computational models of pancreatic organoids shed light on size oscillations in early phases of organoid growth, as well as on the molecular mechanisms responsible for their subsequent branching morphogenesis. 

### 3.4. Models of Neural Organoids

Neural organoids encompass a family of organoid types named after the anatomic regions they model, such as retinal organoids, cerebral cortical organoids, telencephalic organoids, hypothalamic organoids, or spinal cord organoids [57]. Despite the tremendous success of neural organoids in modeling nervous system development and disease, relatively few computational models have emerged so far. 

Eiraku et al. [58] built organoids that mimicked a key step in eye development: the transition from the optic vesicle (an outgrowth of the developing forebrain) to the optic cup (an epithelial structure resembling a double-walled cup, whose external layer gives rise to the pigmented epithelium, whereas the internal one becomes the neurosensory retina). Their paper initiated the vast field of retinal organoid research [59]. However, despite remarkable progress, it took years to elucidate the mechanical signaling involved in optic cup organoid formation, and computational modeling played a central role in it [60]. 

**Figure 2:**
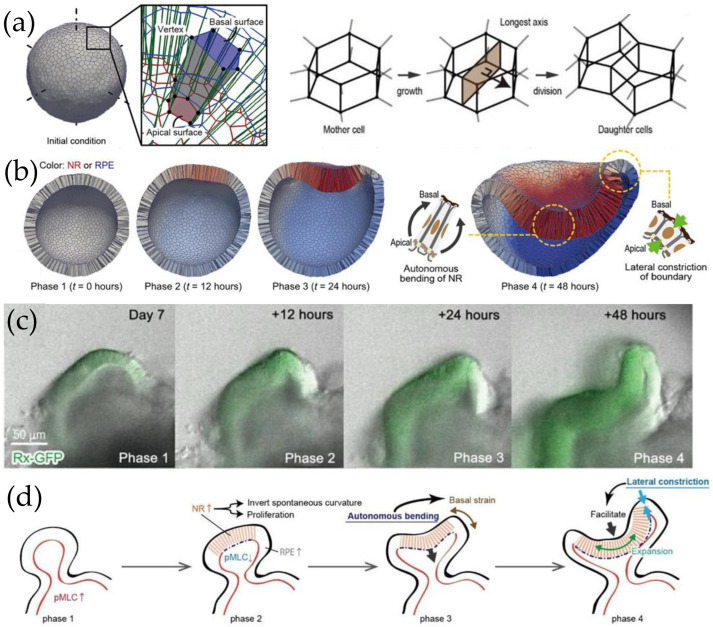
Vertex model simulations of optic cup formation. (**a**) The in silico model of the optic vesicle (left); the representation of cells by polyhedra defined by vertices and edges (middle); schematics of the implementation of cell division in the vertex model (right). (**b**) Simulation snapshots of multicellular self-organization during optic cup formation shown in cross-sectional view. (**c**) Time-lapse fluorescent microscopy images of in vitro optic cup formation (here, Rx-GFP expressing cells appear in green). (**d**) Conceptual model of optic cup morphogenesis. (Adapted with permission from [60], under the terms of the Creative Commons Attribution NonCommercial (CC BY-NC) License 4.0.).

Okuda et al. [60] simulated optic cup formation within the framework of a 3D vertex model—a boundary-based model that represents cells as polyhedrons and describes the tissue as a contiguous network composed of the vertices and edges of the constituent polyhedrons (Figure 2a). The total energy of the system depends on individual cell properties as well as on their interactions with their surroundings. Each vertex moves under the action of the force that acts on it, given by the negative gradient of the total energy at the position of the vertex [61]. In the optic cup model, the total energy contains the sum of cell surface energies and elastic energy terms that are minimal for cells that have a target volume, height, basal surface area, and apical perimeter length. Cell–cell interactions are considered proportional to the lengths of apical edges (where most of the N-cadherins are located in the optic cup cultured in vitro [58]). Model parameters were taken from the literature, if available; otherwise, they were varied systematically to reach agreement with in vitro optic cup morphologies [60]. 

The simulations reproduced experimentally observed shape changes at the individual cell level, predicting the emergence of the apically convex neural retina and the sharply curved hinge structure at the boundary that separates the neural retina from the pigmented epithelium (compare Figure 2b,c). Moreover, the model accurately reproduced cell morphologies in each region: columnar in the neural retina, cuboidal in the pigmented epithelium, and wedge-shaped at the boundary. Combined in vitro and in silico experiments yielded the following conclusions (Figure 2d). The neural retina invaginates on its own by reducing actomyosin accumulation on its apical side. The bending generates strain on the basal surface of boundary cells; mechanosensing leads to calcium influx, which induces their lateral constriction. Thus, the bending rigidity of the border region decreases sharply, facilitating the bending of the neural retina [60]. 

The Biological Cellular Neural Network Modeling (BCNNM) framework, developed by Bozhko et al. [62], was designed to simulate neural organoid growth, axon and dendritic tree formation, axon growth and pathfinding, and synapse formation. The capabilities of the model were illustrated by simulating the self-organization of a four-layered cerebral organoid comprising over 800,000 cells, among which about 130,000 neurons connected by millions of synapses [62]. 

The patterning of human neural tube organoids (NTOs) was investigated by Abdel Fattah et al. in a combined experimental and computational study [63]. They established NTOs from single human induced pluripotent stem cells cultured within a polyethylene glycol hydrogel supplemented with laminin. When treated with retinoic acid and smoothened agonist, about 60% of the NTOs developed a floor plate (FP) domain, whose patterning was visualized using a fluorescent marker (FOXA2). FP expression was mainly scattered, but in 34% of the cases, starting from day 7 in culture, FP cells rearranged, giving rise to a patterned phenotype. The authors built a computational model of the organoid’s cross-section, consisting of interconnected cells on a circle, whose evolution was described by a neighborhood watch algorithm. In their model, a simulation starts with randomly distributed source cells, which release a fate-activating, diffusible signaling molecule (e.g., sonic hedgehog secreted by FP cells). Wherever the signaling molecule’s concentration exceeds the activation threshold, cells adopt or retain the FP fate; outside such favorable zones, they become or remain of non-FP identity. The model suggests that epithelial patterning depends on the initial positions of source cells and morphogen diffusion characteristics, and its predictions were found to agree with experimental results [63]. 

Despite the impetuous development of neural organoid research, relatively few computational models of neural organoids are available to date, and they are highly specialized, with no overlap. Nevertheless, within their narrow focus, they contributed to a deep understanding of the experimentally observed organoid behaviors. 

### 3.5. Model of Kidney Organoids 

Human kidney organoids were obtained via directed differentiation of pluripotent stem cells on a Matrigel bed, in suspension culture, and in hydrogels [64,65]. Unlike epithelial organoids, they are composed of a compact cell mass that comprises nephrons, collecting ducts, renal interstitium, and endothelial cells. Their gene expression profile closely resembles that of the first-trimester human fetal kidney [64]. Nerger et al. investigated the differentiation of human kidney organoids embedded in alginate hydrogels of different stiffness and viscoelasticity [65]. To infer the hierarchy of factors that regulate nephron morphology and patterning, they built a 2D computational model of kidney organoids. Therein, cells are viewed as elastic spheres whose motility, growth, and proliferation are modulated by the pressure exerted by their neighbors. If the pressure is less than critical, a model cell can establish linkages with up to two nearest neighbors, provided that the speeds of the cells to be linked are lower than a threshold. Linkages are considered irreversible and behave like an elastic spring. In the model, a chain of linked cells represents a nephron segment. The simulations indicated that organoids embedded in a barely deformable elastic matrix are unable to form nephron segments. Hydrogels with different rates of deformation (stress-relaxation) induced different nephron patterning: for slow relaxation, nephron segments were confined to the organoid’s surface, whereas for fast relaxation, they had a more uniform distribution. Taken together, experiments and computer simulations suggest that, besides biochemical signaling, the mechanical properties of the encapsulating medium regulate nephrogenesis in kidney organoids [65].

### 3.6. Models of Inner Cell Mass Organoids

In mammalian development, a crucial phase is the evolution of the fertilized egg into a blastocyst able to implant itself into the endometrium. The blastocyst consists of an outer layer of cells (trophectoderm) that encloses a fluid-filled cavity and a cluster of cells known as the inner cell mass (ICM). Aiming to characterize the differentiation of ICM into embryo precursors (epiblast, Epi) and yolk sac precursors (primitive endoderm, PrE), Mathew et al. established murine ICM organoids starting from the reaggregation of about 200 embryonic stem cells [66]. 

In the early blastocyst, ICM cells express the transcription factors NANOG (N) and GATA6 (G). The PrE specification is associated with G expression and the downregulation of N, whereas the Epi specification proceeds in the opposite direction. ICM organoids recapitulate in vivo phenomena. Initially, they contain N_+_G_-_ cells (i.e., cells with high N and low G expression), N_-_G_+_ cells, N_+_G_+_ cells, and N_-_G_-_ cells. After one day in culture, only N_+_G_-_ and N_-_G_+_ cells remain in the organoid, and within one more day, they segregate into a spheroid of N_+_G_-_ wrapped in a sheet of N_-_G_+_ cells. At this stage, laminin is secreted by N_-_G_+_ cells on the outer surface of the organoid, whereas by day 3, laminin is also present between the N_-_G_+_ cells and the N_+_G_-_ cell core. Apart from containing about 15 times more cells, the proportions and spatial arrangements of the cells present in ICM organoids on day 2 are in good agreement with the mid-blastocyst stage [66]. 

Liebisch et al. built an in silico model of ICM organoids to test the hypothesis that the local cell fate clustering observed after one day in culture stems from cell division (and does not involve chemical signaling or cell sorting), provided that the initially specified cell fates are passed on to both daughter cells [67]. 

In their off-lattice, center-based model, cell–cell interactions are described by a Morse potential truncated at two cell diameters. The cell radius is assumed to grow over time up to a maximum value at which the cell divides; the radii of the daughter cells are determined from volume conservation. Cell fate heredity is described by a kinetic model whose parameters are determined by comparison with in vitro data.

The simulation commenced with 200 undifferentiated cells to mimic the in vitro conditions. They proliferated until the cell number increased by 50%. At this point, the initial cell fate decision took place by randomly assigning one of the four expression types: N_+_G_+_, N_+_G_-_, N_-_G_+_, or N_-_G_-_. Then, local cell clusters formed because of cell proliferation. 

The cell proportions and arrangement observed in one-day-old ICM organoids were found to be compatible with the assumption that cell fate is passed on to both daughter cells. Nevertheless, it was necessary to consider cell fate switches to ensure agreement with the cell proportion data of day 2. The neighborhood statistics, however, were still poorly approximated by the model, and the authors suggested that cell sorting driven by differences in cell surface fluctuations [68] could be responsible for the patterning observed on day 2 [67]. 

### 3.7. Models of Tumor Organoids

Early models of tumor organoids focused on epithelial acini and their cancerous lesions. Epithelial acini are lumen-enclosing monolayers, which serve as in vitro models of cysts and tubules, the building blocks of epithelial organs (e.g., mammary glands, kidneys, etc.) [69]. Grant et al. built a 2D computational model to simulate normal and abnormal epithelial cell morphogenesis [70]. In their model, the biological system is represented in cross-section on a hexagonal lattice whose sites are occupied by individual cells, matrix, or free space. Simulations are based on a set of axioms inferred from wet lab experiments. The axioms were adjusted stepwise to ensure a good agreement in vitro behavior. When certain axioms were modified or inconsistently applied, the simulated growth patterns were similar to cancerous lesions [70]. The axioms were further refined by Kim et al. [71] to ensure that simulations produce convex cystic structures in embedded culture, and in a subsequent work, the authors explored the consequences of dysregulations of certain axioms [72]. Engelberg et al. [73] extended the CompuCell3D open-source software package [74,75] to incorporate cell behaviors observed during cyst formation by Madin-Darby canine kidney (MDCK) cells grown in Matrigel. Their in silico model was built on a 2D lattice and allowed for simulating changes in cell size and shape, polarization, and specific orientation of cell division. The model replicated in vitro findings and revealed, among other findings, that lumen formation is not conditioned by the apoptosis of cells that lose contact with the matrix [73]. 

When individual cells of the MCF10A breast cell line are cultured on Matrigel, they proliferate and form clusters within 5-6 days and then self-organize into acini [76]. Rejniak and collaborators simulated the in vitro formation of mammary acini using their 2D biomechanical model of acinar cross sections [77,78,79]. They sought to identify the biomechanical and molecular mechanisms responsible for cell polarization, lumen formation and maintenance, cessation of acinar growth, and stabilization of the acinar structure. In their immersed boundary cell (IBCell) model, cells are described as viscous fluid droplets enclosed in an elastic membrane, whereas their medium is considered a fluid of different viscosity. Fluid movement is described by numerically solving the Navier–Stokes equations, whereas the cytoplasmic membrane and its underlying actin cortex are viewed as a chain of massless point particles connected by springs. A cell boundary interacts with other cell boundaries and the adjacent fluid media (i.e., model cells are endowed with adhesivity and viscoelasticity) [80]. The model revealed that acinar development depends on the concerted action of outer, inner, partially polarized, and fully polarized cells. Their growth, division, epithelial polarization, and apoptotic death eventually lead to the formation of normal hollow acini. Changes in model parameters and assumptions resulted in degenerate acini. Further details on the predictions of the IBCell model are provided in excellent review papers [81,82]. 

**Figure 3:**
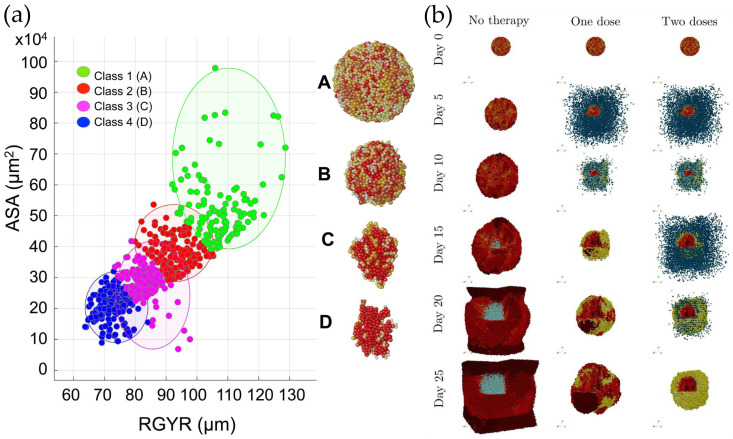
Cell-center models of tumor organoids. (**a**) Simulated organoids classified by an unsupervised machine learning algorithm (k-medians) according to their radius of gyration (RGYR) and accessible surface area (ASA): spherical (Class 1, A), compact (Class 2, B), elongated (Class 3, C), and branched (Class 4, D). Representative members of each class are shown on the right side of the scatter plot, in colors coding different stages of the cell cycle: pearl G1, yellow S, orange G2, carmine M, imperial red cell-cycle arrest [83]. (**b**) Simulation results illustrating the impact of CAR T-cell therapy on a tumor organoid [84]: no therapy (**left**), after a single dose (**middle**), and after two doses (**right**) of CAR T cells, represented by dark blue spheres. The tumor organoid is assumed to be heterogeneous, composed of four cell types that differ in their oncoprotein expression: high (carmine), intermediate (imperial red), low (orange), and very low (yellow)—the latter is insufficient to render the cancer cell recognizable by CAR T cells. Cyan spheres represent dead cells [84]. (Adapted with permission under the terms of the Creative Commons Attribution (CC BY) License 4.0.).

Recognizing the need for a detailed morphometric analysis of tumor organoids, Karolak et al. elaborated a center-based 3D model to simulate the growth of tumor organoids up to 150 μm in diameter [83]. The size limitation was imposed to avoid hypoxia and limited nutrient access in the central region of the organoids. In their lattice-free model, referred to by the acronym MultiCell-LF, a cell is represented by its centroid. It has a radius that increases as the cell progresses through its cell cycle, with most of the growth occurring during the G1 phase. The model accounts for contact inhibition by halting the proliferation of a cell when the number of its neighbors exceeds a threshold. If the centroids of two neighboring cells are closer than the sum of their radii, they repel each other by an elastic spring force; if they are within 2 and 2.25 times the maximum cell radius, they attract each other by another spring force, representing cell adhesion; otherwise, they do not interact. Cellular rearrangements are simulated in the overdamped limit, when the centroids approach their equilibrium positions smoothly, without oscillations [83]. The authors quantified the morphology of in silico tumor organoids obtained for various model parameters (cell radius, cell division age, and sensitivity to contact inhibition). For example, strict contact inhibition resulted in irregular shapes, whereas mild contact inhibition favored the spherical configuration. The authors argued that drug exposure depends on the organoid’s morphology and introduced two parameters to quantify it. The accessible surface area (ASA) was defined in analogy with the ASA of proteins. Compactness was characterized by the radius of gyration, defined as the mean square distance of individual cells from the organoid’s center of mass. Figure 3a shows the results of k-medians clustering of simulated tumor organoids and depicts a representative member of each class (A-D). In therapy, the authors suggest considering not just tumor size but also its morphometric data, as they may influence the organoid’s drug exposure and drug penetration [83]. 

Patient-derived organoids comprise diverse cell populations, which makes them difficult to eradicate. Luque et al. [84] looked at the impact of tumor heterogeneity on the outcome of chimeric antigen receptor (CAR) T-cell therapy. They tested different treatment strategies to identify one that is effective at relatively low doses of CAR T cells (i.e., with minimal side effects). They built a center-based computational model of cancer cells and immune cells evolving under the action of adhesive, repulsive, active, and drag forces [85]. To account for intratumor heterogeneity, in the model, cancer cells are supposed to express different (normally distributed) levels of a mutant oncoprotein that determines both their proliferation rate and immunogenicity (Figure 3b). Additionally, cancer cells are assumed to secrete an immunostimulatory factor that diffuses and induces CAR T cell chemotaxis. Upon adhesion to a cancer cell, a CAR T cell determines its apoptosis with a probability proportional to its level of oncoprotein expression. If successful, it detaches and undergoes a biased random walk towards other cancer cells; if not, it remains attached and attempts to kill its partner; after a maximum attachment lifetime, the immune cell aborts its mission and migrates away [84]. 

The simulations uncovered several interesting phenomena. First, they demonstrated that treatment efficacy does not increase monotonously with CAR T cell dosage. Second, they showed that increasing the persistence time (the time until CAR T cells become exhausted) from 10 days to 30 days does not render small-dose treatments more effective. Large doses, on the other hand, can result in a large percentage of free CAR T cells, which can lead to side effects. Third, fractional dosing improved the treatment outcome, but the second dose was less effective than the first. Remarkably, after the second dose, cancer cells with very low oncoprotein expression covered the organoid’s surface, shielding other cancer cells (Figure 3b—right column). Finally, multi-antigen recognition CAR T-cell therapy was found to eliminate the organoid if the number of immune cells was at least 50% larger than the number of cancer cells, but concerns remain regarding their potential side effects in a clinical context [84]. 

Let us take a retrospective look at this subsection. Since most cancers originate from epithelia, computational models first focused on cellular processes involved in cyst formation in vitro and alterations thereof, uncovering potential paths to precancerous lesions. The emergence of patient-derived tumor organoids motivated the development of their in silico counterparts, and computer simulations revealed physical and biological factors that could affect therapy outcomes. 

### 3.8. Generic Organoid Models 

Many types of organoids consist of an epithelial cell sheet enclosing a fluid medium, yet they adopt a variety of shapes, including spherical, budded, branched, or invaginated structures [22]. In their quest for the physical basis of small-organoid morphogenesis, Rozman et al. constructed a 3D, surface tension-based, active vertex model of a closed epithelial cell sheet composed of a single cell type [86]. Cell differentiation was neglected on purpose to highlight the implications of cell-level mechanics, such as the apico-basal differential surface tension. 

In their model, cells are represented as polyhedra with polygonal apical (inner) and basal (outer) faces and rectangular lateral sides shared with their neighbors; they are assumed to be incompressible and feature three distinct surface tensions (apical, lateral, and basal). Cell rearrangements are ascribed to vertex movement driven by energy minimization and active junctional noise. The latter mimics the stochastic dynamics of molecular motors, endowing the model tissue with viscous fluid-like behavior [87]. Despite its simplicity, the model could reproduce several organoid morphologies and provided surprising insights. For example, it revealed that the formation of branched epithelial structures requires intense junctional activity and demonstrated that the modulation of cell height does not hinge on cell differentiation but can result from apico-basal polarity alone [86]. 

Tanida et al. tackled the problem of organoid morphogenesis in the framework of a phase-field model [88]. Therein, field variables represent the lumen, as well as individual cells that adhere to each other. The evolution of each cell is described by a modified Allen-Cahn equation that includes a force term accounting for cell–cell adhesion, excluded volume interaction, and cell growth. Cell division is assumed to occur after a certain time, provided that the cell volume has reached a predefined value; otherwise, the division takes place later, when the volume criterion is met. In addition to cell dynamics, the model also describes the time course of the lumen. Computer simulations predicted the formation of monolayer morphologies (cysts and branched structures) and multilayer configurations (branched, single lumen, multiple lumens, no lumen). In particular, the model pinpointed the key conditions that ensure the formation of spherical organoids made of an epithelial cell monolayer that wraps a fluid-filled lumen [88]. 

Among the many factors that influence organoid growth, vascularization and the availability of oxygen and nutrients are critical, especially when the organoid comprises several layers of cells (e.g., tumor and kidney organoids). Organoid-on-a-chip platforms use microfluidics to control fluid flow, nutrient supply, and waste removal, creating a dynamic and physiologically relevant microenvironment that enhances organoid development and function. This context is addressed by the computational model conceived by Carrasco-Mantis et al. [89]. The model features a generic organoid, akin to a multicellular spheroid, partially embedded in a hydrogel, with its free surface exposed to the flowing cell culture medium. In addition to organoid growth, it considers vascular network formation on the organoid surface as a function of the shear stress caused by fluid flow. The organoid is represented within an agent-based model inspired by the Chaste platform [90], whereas vasculogenesis is described along the lines developed by Perfahl et al. [91]. The Navier–Stokes equations characterize fluid flow, and reaction–diffusion equations describe the transport and consumption of oxygen and nutrients; all these equations are solved numerically using the finite element method. Model predictions have been validated against experimental data on kidney organoids cultured under flow in millifluidic chips [92]. Nevertheless, given the flexibility of this hybrid computational model, it might be adapted to study the evolution of vascularized tumor organoids, as well [93,94].

Table 1 provides a synthesis of the papers included in this review. 

## 4. Discussion

Computational models of organoids have attracted increasing interest recently. They are viewed as cost-effective tools that can uncover molecular mechanisms and physical factors involved in organoid growth. Thereby, they can contribute to the optimization of organoid cultures. 

This vibrant research field has been reviewed previously. Montes-Olivas et al. provided an overview of available computational models of organoids and discussed the strengths and limitations of computer simulation frameworks that have been or could have been used for organoid modeling [25]. Poli et al. focused on neural organoids and discussed computational tools that might be applied to study them in silico [26]. In their expert opinion paper, Norfleet et al. analyzed the requirements that need to be fulfilled by computational modeling software to be applicable in organoid research [27]. They also discussed experimental methods and biophysical principles that can guide model development. Finally, the narrative review of Thalheim et al. [29] is an in-depth analysis of insights gained from computational models of organoids, and a thorough discussion of the field’s perspectives. 

This article aims to provide a holistic image of agent-based computational studies of organoids and to serve as a catalyst for collaborations between research teams focused on diverse aspects of organoid biology. In our opinion, the know-how exchange is utterly needed because, even for a given organoid type (e.g., intestinal), current modeling efforts (Table 1) involve a variety of computational techniques, depending on the investigated problem and available hardware. 

Researchers can identify common features encountered in different approaches and suggest optimal implementation strategies, but perhaps they should not aim at building a gold-standard model for each organoid type. Experts suggest that a detailed in silico representation of organoids is neither desirable nor feasible [29]. By increasing model complexity, one raises the demand for computational power and makes the implementation more error-prone. What is more important, high complexity would blur the direct insight into the dominant mechanisms of tissue self-organization [29]. 

Computational models developed so far have explained specific features of organoid cell composition, growth, shape, and dynamics. Moreover, they provided working hypotheses for in vitro experiments. For example, simulation-based insights clarified several aspects of intestinal organoid biology. Three distinct computational models of organoid budding and intestinal crypt fission considered Paneth cells to be more adhesive than intestinal stem cells [36,39,40], pointing out the central role of integrins in the formation of new crypt-like domains. Buske et al. also suggested that Paneth cell differentiation hinges on high Wnt signaling, which in turn might depend on integrin-linked kinase activity [36]. Subsequent research provided some support for the involvement of integrin-linked kinase in Wnt signaling [95]. But other molecular mechanisms have also been explored, and a growing body of evidence indicates that integrin binding to the ECM does modulate Wnt signaling [96]. The activation of focal adhesion kinase (FAK) due to integrin ligation was found to synergize with the Wnt pathway via the adaptor protein Grb2 [97]. The apical net considered by Thalheim et al. [37] in their simulations of organoid growth patterns favored budding by reinforcing cell–cell interactions on the apical side of the epithelium. This finding is in agreement with the analytical theory of Yang et al., which predicts that budding is mainly initiated by the apical constriction of crypt-forming cells [47]. 

If in vitro organoid research benefited from computer simulations, the reverse is also true. Experimental data on signaling pathways that control stem cell self-renewal and lineage commitment were incorporated into informative computational models. Next-generation in silico models of organoids are expected to also account for the crosstalk of various signaling pathways and the molecular mechanisms involved in the biomechanical regulation of stem cell fate. Promising directions of model development along these lines are discussed by Thalheim et al. [29]. 

In the short term, we will most likely witness a diversification of cell-based computational models because certain organoid types have not yet been investigated by such tools. For example, human heart organoids have gathered momentum recently [98,99,100,101], but the modeling community focused more on the functional than the structural features of cardiac organoids [102,103]. 

This review also aimed to highlight connections between the early stages of 3D biology and modern organoid research. For instance, the organoids discussed in the first two paragraphs of Section 3.7 were not derived from stem cells, but from immortalized cell lines. Although they lack the complexity of present-day organoids grown from tumor biopsies, they have provided invaluable insights into cancer biology, such as the discovery of the bidirectional cross-modulation of β1-integrin and epidermal growth factor receptor (EGFR) signaling, which enabled the restoration of the normal breast cell phenotype by down-modulating EGFR in certain tumor cells [104]. Moreover, the computational models of those organoids are excellent starting points for in silico models of patient-derived tumor organoids. 

Computational organoid models can boost their predictive power by applying machine learning for the analysis of simulation results and experimental data. For example, the clustering of simulation results shown in Figure 3a was performed using the k-medians algorithm, an unsupervised machine learning algorithm designed to group a set of data into a given number of distinct clusters. It is especially instrumental when outliers are present in the data, as they can skew the mean. The authors argued that grouping organoids into distinct morphophenotypic classes can serve clinical practice by predicting a tumor’s sensitivity to chemotherapy [83]. To characterize the patterning of ICM organoids, Dirk et al. [105] used a graph neural network trained with synthetic data generated by a mathematical model of cell–cell communication [106]. Then, they applied the graph neural network to the in vitro data obtained by Mathew et al. on ICM organoids [66], and concluded that the experimentally observed pattern was consistent with short-range cell–cell communication: the fate of a given cell could be predicted with 70% accuracy from the fates of its nearest neighbors [105]. 

Machine learning and deep learning techniques have revolutionized the processing of organoid images. Traditional imaging methods have been deemed limited by the complexity of organoid phenotypes, focusing issues, and large data volumes. To address these challenges, Borten et al. developed OrganoSeg, an open-source software that enables segmentation, filtering, and analysis of brightfield images of 3D spheroids and organoids [107]. The need for human intervention was minimized in OrgaQuant, a software built by Kassis et al. for automatic detection and quantification of intestinal organoids based on brightfield images. OrgaQuant uses a convolutional neural network (CNN) based on the Faster R-CNN architecture, implemented in TensorFlow [108]. Gritti et al. built MOrgAna, a user-friendly image analysis pipeline implemented in Python 3.9 for high-throughput analyses of organoid images [109]. In MOrgAna, segmentation is performed on bright-field images using algorithms such as Logistic Regression and Multi-Layer Perceptron. The growth of tumor organoids was monitored over time by Branciforti et al. using optical coherence tomography (OCT), a non-invasive and label-free technique; the acquired 3D images were analyzed by a deep learning algorithm based on the K-Net architecture with a Swin Transformer backbone [110]. In the study conducted by Ong et al., the 3D structure of organoids was quantified by artificial intelligence tools bundled with a user-friendly interface called 3DCellScope [111]. Their workflow includes fast 3D segmentation, analysis of topological descriptors, and generation of morphological signatures. To this end, one needs stacks of fluorescent microscopy images of the organoid, with actin staining for cell borders and DAPI staining for nuclei. Nucleus and cell segmentation tasks are performed by DeepStar3D, a pre-trained CNN based on StarDist principles, providing the user with an accurate 3D digital replica of the organoid, characterized by hundreds of morphological and topological descriptors. These reveal tissue patterns and facilitate the assessment of spatial relationships and mechanical constraints [111]. Quantitative data retrieved from organoid imaging can be used for model validation. 

Future progress in the field of in silico organoid models may also come from insights gained from computational studies of biological tissue dynamics observed in development and disease [16,112,113,114]. 

For over three decades, the cellular Potts model contributed to our understanding of multicellular self-organization. It is implemented in powerful open-source software packages suitable for multiscale simulations, CompuCell3D [75] (https://compucell3d.org/, accessed 29 November 2025), and Morpheus (https://morpheus.gitlab.io/, accessed 29 November 2025). CompuCell 3D was employed by Tikka et al. to explore early nephrogenesis [115]. They demonstrated that nephron progenitor cells can form proximal tubular aggregates via chemotaxis and differential cell adhesion. Simulation results were validated by experiments conducted on kidney organoids built by dissociation and reaggregation [115]. Alsubaie et al. investigated epithelial tissue invasion using CompuCell3D [116]. In simulations of the competition between adjacent epithelial cell populations, they found that invasion can originate from differences in cell adhesivity or cell perimeter contractility, despite the two populations having the same rates of cell proliferation and cell death. Such simulations, performed in a 3D configuration akin to patient-derived tumor organoids [20] could potentially characterize population dynamics in histologically complex and genetically heterogeneous tumor organoids. Within the same computational environment, Pramanik et al. studied ECM remodeling during cancer invasion [117]. Thus, the cellular Potts model holds promise to shed light on organoid growth, as well (see, e.g., [73]).

PhysiCell is another open-source multicellular simulator with well-established predictive power [118] (https://physicell.org/, accessed 29 November 2025). It describes multicellular dynamics based on physical principles and includes advanced cell cycle models and solvers for reaction–diffusion equations. PhysiCell was applied to simulate the in vivo process known as ductal carcinoma in situ, the earliest form of breast cancer, in which clusters of abnormally dividing cells of epithelial origin are found within the milk ducts [118]. The same biological process was simulated about 150 times faster by Du et al. in Gell (https://github.com/PhantomOtter/Gell, accessed 29 November 2025), their multicellular simulation environment based on graphics processing unit (GPU) computing [119]. Also, PhysiCell was combined with Boolean modeling of intracellular signaling in an open-source software called PhysiBoSS [120] (https://github.com/sysbio-curie/PhysiBoSS, accessed 29 November 2025), which proved to be effective in replicating experimental results on cancer cell invasion, and holds promise to anticipate the risk of metastasis in patients with certain mutations [121]. 

Many types of organoids share an epithelial structure [4]. Therefore, vertex models have been proposed as the most appropriate framework for describing the shape changes and collective motion of cells embedded in a monolayer, and accounting for mechanical feedback on lineage specification [29]. This review analyzed several applications of vertex models in organoid research, and the sustained interest in this field [61,122,123,124,125,126,127,128,129] suggests that more are expected to emerge. 

The Open Virtual Tissues (OpenVT) initiative (https://morpheus.gitlab.io/tag/open-virtual-tissues/, accessed 30 November 2025) aims to facilitate the development of modular, reproducible, and reusable simulations. In expert meetings and annual competitions, it seeks to identify solutions for building multicellular models that are findable, accessible, interoperable, and reusable (FAIR; see https://www.go-fair.org/fair-principles/, accessed 30 November 2025). It is recommended to make in silico tissue models available by uploading them to a public repository such as BioModels (https://www.ebi.ac.uk/biomodels/, accessed 30 November 2025)—see, e.g., MODEL2503030003, Luque et al. [84]. 

This systematic review provides a state-of-the-art overview of cell-based computational models of organoids, examining their current applications, impact, and future directions. While readers will ultimately assess the strengths of this work, it is important to acknowledge its limitations. The first, and perhaps most important limitation, is its reliance on only three major databases (PubMed, Scopus, and Web of Science). Therefore, relevant papers might have been missed, so this review is not comprehensive. We apologize to the authors whose work was not included. Second, this review was limited to peer-reviewed articles. We chose not to include the grey literature because it did not pass the filter of peer review. This, however, could have resulted in publication bias and a less up-to-date review. 

## 5. Conclusions

Computational models of organoids are diverse. Most of them are specifically tailored for a particular type of organoid and aim to address a well-defined question. Several examples discussed in this article demonstrate that computer simulations can offer profound insight into the physicochemical mechanisms that govern organoid growth. Computational studies proved to be most effective in combination with experiments designed to infer model parameters and/or validate model predictions. 

The perspectives of in silico organoid research may be strongly influenced by progress in systems biology, the use of machine learning in organoid imaging, and the development of modular software for multiscale simulations. Such software should account for the crosstalk of regulatory pathways of stem cell fate, nutrient and oxygen exchange, cell–cell and cell–ECM interactions, cell motility, and ECM remodeling. 

## Figures and Tables

**Table 1:** Overview of cell-based computational models of organoids.

Reference	Organoid Type	Model Type ^1^	Modeled ECM	Cell Proliferation and Differentiation	Main Predictions
Buske et al. [36]	intestinal	elastic sphere, 3D	basement membrane	Wnt and Notch signaling; Paneth cell specification depends on the monolayer’s curvature	The basement membrane is remodeled by the cells it hosts. Biomechanics modulates stem cell fate. Crypt-like domains are initiated by shape fluctuations caused by cell proliferation.
Thalheim et al. [37]	intestinal	elastic sphere, 3D	basement membrane and apical net	Wnt and Notch signaling; Paneth cell specification depends on tissue curvature	The model replicates the experimentally observed branched and cyst-like phenotypes. Geometric transformation accounts for cell shape changes observed in cyst-like organoids.
Pin et al. [38]	intestinal	rigid sphere, 3D	basement membrane	Wnt and Notch signaling	The model replicates the cell population dynamics of intestinal crypts.
Pin et al. [39]	intestinal	deformable sphere, 3D	basement membrane	Wnt and Notch signaling	Crypt fission is simulated based on the assumption that proliferative cells behave as Newtonian fluids, whereas Paneth cells deform as Bingham plastics.
Langlands et al. [40]	intestinal	cell-center, 2D	embedding Matrigel	stochastic cell cycle model, fixed probability of asymmetric division	In Paneth-cell-rich regions of an intestinal organoid, stem cells are pushed out of the monolayer, initiating the formation of new crypt-like domains.
Almet et al. [44]	intestinal	cell-center, 2D	embedding Matrigel	stochastic cell cycle model, fixed probability of asymmetric division	Budding and crypt fission occur because Paneth cells are stiffer than stem cells and adhere more strongly to the adjacent ECM.
Montes-Olivas et al. [45]	intestinal	cell-center, 2D	embedding Matrigel	stochastic cell cycle model, fixed probabilities for stem cells to give rise to Paneth cells or transit-amplifying cells	The model reproduced the average number of crypts per organoid observed in vitro on days 5 and 7 in culture. Also, the circularity of the simulated organoids was like that of lab-grown organoids.
Yang et al. [47]	intestinal	vertex, 3D, analytical	-	-	The model revealed that bud formation is driven by differential spontaneous curvature. It also explained why lumen inflation leaves budded crypts unchanged.
Laussu et al. [48]	intestinal	active vertex, finite element model, 3D	-	-	The shape changes of human colon organoids were simulated under diverse mechanical loads. The strain and stress distributions were mapped at subcellular resolution.
Elosegui-Artola et al. [49]	intestinal	elastic sphere, 2D	embedding hydrogel (alginate-Matrigel interpenetrating networks)	constant cell division rate modulated by a Monte Carlo algorithm to discourage energetically unfavorable proliferation	In an elastic ECM, intestinal organoids grow slowly and maintain a spherical shape; in a viscoelastic ECM, they grow rapidly and undergo symmetry breaking followed by finger formation. Similar mechanisms might be involved in embryonic airway branching, wound healing, and tumor invasion.
Larrañaga et al. [50]	intestinal	elastic sphere, 3D	Matrigel-coated substrate with dots of exogenous Wnt	Wnt and BMP signaling	The model reproduced the experimentally observed compartmentalization of the monolayer into crypt- and villus-like domains. When Wnt dots were 200 μm apart, the distribution of crypts was congruent with the Wnt dots; at a smaller/larger spacing, crypts were fewer/more than Wnt dots.
Pérez-González et al. [52]	intestinal	vertex, 3D	Matrigel substrate, soft and hard	-	In intestinal organoids grown on soft or hard substrates, the crypt-like domain is shaped by the apical constriction of stem cells and collective cell migration.
Sachs et al. [53]	airway	cell-center, 3D	-	-	The collective rotational movement of cells in airway organoids stems from cell–cell communication that tends to align a cell’s active traction force with the total force that acts on it.
Hof et al. [55]	pancreatic	cell-center, 3D	-	cell division at a rate assessed by light sheet microscopy	The smaller an organoid, the more prone it is to undergo size oscillations due to ruptures caused by osmotic influx. The frequency of ruptures depends on the cell proliferation dynamics.
Dahl-Jensen et al. [56]	pancreatic	cubic lattice, 3D	-	cell division regulated by signaling molecules produced by cells	The experimentally observed branching morphology was replicated by the model under the assumption that the signaling molecule released by the cells is a dimer- or trimer-forming inhibitor.
Okuda et al. [60]	optic cup	vertex, 3D	-	cell proliferation model that describes cell growth and division	Optic cup formation was simulated by the model both at tissue and individual cell level. Modeling indicated that the bending of the neural retina is caused by the lateral constriction of the cells located at its boundary (next to the pigmented epithelium).
Bozhko et al. [62]	neural	cubic lattice, 3D	-	stochastic model of cell proliferation	The model describes the growth of a cerebral organoid of up to 10^6^ cells and simulates axon guidance by chemical signaling, as well as the formation of synapses.
Abdel Fattah et al. [63]	neural	cell-center, 1D	-	cell differentiation takes place when the morphogen concentration exceeds an activation threshold	Floor plate (FP) domain patterning in human neural tube organoids results from randomly distributed source cells that secrete a morphogen with appropriate diffusion characteristics. FP inhibition via WNT and bone morphogenic protein (BMP) signaling can modulate FP patterning.
Nerger et al. [65]	kidney	elastic sphere, 2D	embedding hydrogel (alginate)	cell growth and division modulated by the pressure exerted by their neighbors	Nephron segment patterning in kidney organoids depends on the stress-relaxation rate of the embedding hydrogel. No nephrons emerge in an elastic matrix incapable of stress relaxation.
Liebisch et al. [67]	inner cell mass	cell-center, 3D		cell growth, stochastic cell division and initial cell fate decision, kinetic models of cell fate heredity	Cell fate clusters observed in one-day-old inner cell mass organoids result from cell division and stable cell fate inheritance. Between day 1 and day 2, the neighborhood statistics is also influenced by cell sorting.
Grant et al. [70] Kim et al. [71,72]	acinar, tumor	hexagonal lattice, 2D	volume elements of the ECM and luminal space	proliferation, apoptosis, matrix remodeling, and lumen formation—axiomatic framework	Twelve axioms mimic normal cell behavior, leading to the formation of acinar organoids by Madin-Darby canine kidney cells. Deviations from certain axioms resulted in multicellular configurations that resembled precancerous lesions.
Engelberg et al. [73]	acinar, tumor	square lattice, subcellular resolution, 2D	volume elements of the ECM and luminal space	cell growth, cell shape changes, cell division, apoptosis, matrix remodeling, lumen formation, and tight junction maintenance	Simulations revealed that (i) luminal cell death is not a necessary condition for cystogenesis, (ii) the axis of cell division has a significant impact on lumen size for a given cell number, and (iii) a cell state change is required to simulate the reduced growth rate of mature cysts.
Rejniak et al. [77,78,79]	acinar, tumor	immersed boundary cell, 2D	embedding medium modeled as a viscous fluid	cell growth, division, apoptosis, polarization	Acinar development by human epithelial breast cells relies on the interactions of at least four cell types, with different growth, proliferation, epithelial polarization, and apoptosis behavior. Carcinomas form when cells lose their epithelial architecture and expand into the lumen.
Karolak et al. [83]	tumor	cell center, 3D	-	cell cycle with four phases, contact inhibition	Simulations of tumor organoid growth resulted in different morphologies depending on the rate of cell proliferation and sensitivity to contact inhibition. Morphometric parameters, such as compactness or accessible surface area, might influence the drug-sensitivity of organoids.
Luque et al. [84]	tumor	cell-center, 3D	-	cancer cells: cell cycle, apoptosis, necrotic death, oncoprotein expression, secretion of immunostimulatory factor; CAR T-cells: self-propelled, perform biased random walk along immunostimulatory factor gradient, attempt to induce cancer cell apoptosis, get exhausted in about 10 days	The simulations demonstrate the challenges faced by CAR T-cell therapy of spatially heterogeneous tumors. The impact of the therapy does not increase monotonously with the (number of CAR T-cells)/(number of cancer cells) ratio; for efficacy and minimal side effects, this ratio should be close to 1. Increasing the persistence time of CAR T-cells does not translate into better therapy outcomes. When CAR T-cells are delivered in two doses, about a week apart, after the second dose, cancer cells with small oncoprotein expression cover the organoid surface, protecting the more treatment-sensitive cancer cells.
Rozman et al. [86]	epithelial, generic	vertex, 3D	-	cell growth and division at fixed rates	Experimentally observed shapes of epithelial organoids can be replicated by an active vertex model comprising a single cell type. Branch formation was found to originate from fluctuations of tensions along cell–cell junctions.
Tanida et al. [88]	generic	phase-field, 2D, 3D	-	cell growth, cell division after a certain time, provided that the cell volume reached a threshold	The model pointed out morphogenetic factors that drive shape formation observed in various organoids. Remarkably, the most common configuration of epithelial organoids (a spherical monolayer of cells enclosing a fluid-filled lumen) was found to emerge for a broad range of model parameters (lumen pressure and minimum cell division time).
Carrasco-Mantis et al. [89]	generic	cell center, 3D	volume elements of the ECM	cell division modulated by oxygen concentration, flow-induced differentiation of stem cells into endothelial cells	The model describes phenomena observed in kidney organoids cultured under laminar fluid flow, including organoid growth, vascular network formation, as well as oxygen and nutrient transport and consumption.

^1^ Computational models employed thus far in organoid modeling fall into the following categories: elastic sphere models (i.e., cells are represented by sticky elastic spheres), rigid sphere models (i.e., cells are viewed as hard spheres that can adhere to each other), cell-center models (i.e., cells are considered point-like objects capable of attractive forces that mimic cell–cell adhesion and repulsive forces that mimic excluded volume interactions), immersed boundary cell models (i.e., cells are described as viscous liquid droplets wrapped in an adhesive, elastic membrane), lattice models (in which cells or subcellular elements reside on the nodes of a grid that represents discretized space), phase field models (which describe cells and their environment in terms of scalar fields whose evolution is governed by partial differential equations), and vertex models (i.e., in 2D, cells are represented as polygons characterized by their vertices and edges; interactions cause vertex movement, which results in tissue shape changes and/or cell motility; in 3D, cells are viewed as polyhedra, as shown in Figure 2a).

## Data Availability

This article does not present original data.

## Supplementary Materials

The following supporting information can be downloaded at: https://www.mdpi.com/article/10.3390/cells15020177/s1, Table S1: Boolean expressions of search terms used to identify relevant literature items.
