# Supplementary material for: Cell-Based Computational Models of Organoids: A Systematic Review
Source: Cells. 2026 Jan 19;15(2):177. doi: 10.3390/cells15020177 (PMC12839770; doi:10.3390/cells15020177)
Supplement: Supplementary file 1 [file cells-15-00177-s001.zip › cells-4064947-supplementary.pdf]

cells

Supplementary Materials

of the review paper

Cell-Based Computational Models of Organoids: A Systematic Review

Monica Neagu, Andreea Robu, Stelian Arjoca, and Adrian Neagu

Table S1 lists the logical expressions used in our systematic search for original articles that could satisfy the inclusion criteria. All the search results were scientific papers written in English. Nine of the returned items were preprints. Since they did not undergo peer review and might suffer changes before being accepted for publication, they were not included in this review.

Table S1. Boolean expressions of search terms used to identify relevant literature items

Database (number of hits)	Boolean expression
**PubMed** (315)	(("computational model*"[Title/Abstract]) OR ("in silico"[Title/Abstract]) OR ("computer simulation"[Title/Abstract]) OR ("theoretical model*"[Title/Abstract]) OR ("mathematical model*"[Title/Abstract])) AND (organoid*[Title/Abstract])
**Scopus** (312)	TITLE-ABS-KEY (("computational model*" OR "in silico" OR "computer simulation" OR "theoretical model*" OR "mathematical model*") AND organoid*) AND (LIMIT-TO (DOCTYPE , "ar") OR LIMIT-TO (DOCTYPE , "cp")) AND (LIMIT-TO (LANGUAGE , "English"))
**Web of Science** (276)	TI=(("computational model*" OR "in silico" OR "computer simulation" OR "theoretical model*" OR "mathematical model*") AND (organoid*))<br><br>AB=(("computational model*" OR "in silico" OR "computer simulation" OR "theoretical model*" OR "mathematical model*") AND (organoid*))
